# Navigating the Rapidly Evolving Advanced Urothelial Carcinoma Treatment Landscape: Insights from Italian Experts

**DOI:** 10.1007/s11912-023-01461-x

**Published:** 2023-10-19

**Authors:** Daniele Santini, Giuseppe Luigi Banna, Sebastiano Buti, Luca Isella, Marco Stellato, Michela Roberto, Roberto Iacovelli

**Affiliations:** 1 Medical Oncology A, University of Rome, Policlinico Umberto I, “La Sapienza, Rome, Italy; 2 Portsmouth Hospitals University NHS Trust, Portsmouth, PO6 3LY UK; 3 Faculty of Science and Health, School of Pharmacy and Biomedical Sciences, University of Portsmouth, Portsmouth, PO1 2UP UK; 4 Department of Medicine and Surgery, University of Parma, Viale A. Gramsci 14, 43126 Parma, Italy; 5 Oncology Unit, University Hospital of Parma, Viale A. Gramsci 14, 43126 Parma, Italy; 6 Medical Oncology Department, Fondazione IRCCS National Cancer Institute, Milan, Italy; 7 UOC Oncology A, Department of Radiological, Oncological and Anatomo-Pathological Science, Policlinico Umberto I, “La Sapienza” University of Rome, Rome, Italy; 8 UOC Medical Oncology, Comprehensive Cancer Center, Fondazione Policlinico A. Gemelli IRCCS, Rome, Italy; 9 Department of Translational Medicine and Surgery, Università Cattolica del Sacro Cuore, Rome, Italy

**Keywords:** Expert opinion, Immune checkpoint inhibitors, Multidisciplinary management, Prognostic factors, Targeted therapy, Urothelial carcinoma

## Abstract

**Purpose of Review:**

To discuss recent advances in the treatment of advanced urothelial carcinoma (UC) and how best to incorporate new therapies into clinical practice.

**Recent Findings:**

There have been several recent practice-changing phase 2 and 3 trials of immune checkpoint inhibitors (ICIs), antibody–drug conjugates (ADCs), and targeted agents in advanced UC. Based on data from these trials, ICIs can be used as first-line maintenance therapy in patients who do not progress on platinum-based chemotherapy, second-line therapy for those with progression, and first-line therapy in cisplatin-ineligible patients with PD-L1 expression; ADCs and targeted agents provide later-line treatment options.

**Summary:**

Despite substantial progress in the treatment of advanced UC, there are still many uncertainties, including the optimal treatment sequence for novel agents, and reliable predictive biomarkers to aid in treatment selection. There is also an unmet need for effective treatment options in patients unfit for any platinum-based chemotherapy.

## Introduction

Urothelial carcinoma (UC) is a common malignancy that affects the lining of the urinary tract (i.e. the bladder, upper urinary tract, and urethra) [1, 2]. The bladder is the most common site of UC, accounting for 90–95% of cases; likewise, UC accounts for approximately 90% of bladder cancers [2, 3]. Upper tract UC (UTUC) occurs in 5–10% of cases, and UC rarely occurs along the urethra (< 1%) [1, 4].

Bladder UC and UTUC mainly occur in elderly individuals, share certain histopathological characteristics, and have other risk factors in common, most importantly cigarette smoking [3, 5, 6]. Therefore, bladder UC and UTUC are considered similar entities, and based on clinical trials conducted mainly in patients with bladder UC, the approach to systemic therapy is generally the same, although UTUC has a worse prognosis [4, 6–9]. Up to 25% of patients with bladder UC and two-thirds of those with UTUC present with muscle-invasive disease, which is associated with substantial risk of metastatic spread [4, 10–13]. Although only 12% of patients with bladder UC have regional or distant metastases at diagnosis [14], approximately 50% of patients with localised muscle-invasive disease who undergo curative-intent cystectomy subsequently relapse and develop metastatic disease [13, 15].

Patients with unresectable locally advanced or metastatic UC can be divided into three broad categories: (1) fit for cisplatin-based chemotherapy, (2) unfit for cisplatin (but fit for carboplatin-based chemotherapy), or (3) unfit for any platinum-based therapy [7, 15]. Cisplatin-based chemotherapy is the preferred first-line treatment for inoperable advanced UC, but ≥ 50% of patients are not eligible for cisplatin according to Galsky criteria, which include an Eastern Cooperative Oncology Group performance status (ECOG PS) ≥ 2, creatinine clearance (CrCl) < 60 mL/min, grade ≥ 2 audiometric hearing loss, grade ≥ 2 peripheral neuropathy, and/or New York Heart Association class III heart failure [7, 13, 16–18]. Patients unfit for cisplatin may be able to receive carboplatin-based chemotherapy, but some patients are deemed unfit for any platinum-based therapy, including those with severely impaired PS (ECOG PS ≥ 3) and/or severely impaired renal function (CrCl < 30 mL/min) [7, 19, 20]. As indicated by a median overall survival (OS) of up to 15 months with cisplatin-based regimens and 9 months with carboplatin-based regimens, relapse and progression are very common with platinum-based chemotherapy [21–25].

In the last decade, immune checkpoint inhibitors (ICIs) targeting programmed cell death protein-1 (PD-1) and programmed death ligand-1 (PD-L1), such as atezolizumab, avelumab, nivolumab and pembrolizumab, have become first-line options for platinum-unfit patients with PD-L1-positive tumours, and first-line maintenance or second-line options for platinum-fit patients [7, 15]. Novel treatment options are now also available for later-line treatment, including the targeted therapy erdafitinib, a pan-fibroblast growth factor receptor (FGFR) tyrosine kinase inhibitor [26], and the antibody–drug conjugates (ADCs) enfortumab vedotin and sacituzumab govitecan [7, 15, 27, 28]. Enfortumab vedotin comprises a fully human monoclonal antibody (mAb) against Nectin-4 (a cell adhesion molecule highly expressed in UC) conjugated to monomethyl auristatin E (MMAE, a microtubule inhibitor) [29, 30]. Sacituzumab govitecan is a humanised mAb targeting trophoblast cell surface antigen 2 (Trop-2; a transmembrane glycoprotein highly expressed in UC) conjugated to SN-38, the active metabolite of the topoisomerase-1 inhibitor irinotecan [31].

With the advent of immunotherapy, targeted therapy, and ADCs, the therapeutic landscape for advanced UC has changed considerably in recent years, and optimal treatment strategies are the subject of some debate. To address this, our expert panel developed a series of questions relevant to current treatment options and optimal treatment sequences. In response to these questions, the expert panel have considered the evidence for different treatment strategies and provided their recommendations on how to best incorporate them into the treatment landscape.

## Evidence Acquisition

To identify key literature on which to base the expert panel’s opinions and recommendations, we performed a search of the PubMed database (to February 2023). Search strategies included the following broad terms alone or in combination: “urothelial carcinoma”, “bladder cancer”, “upper tract urothelial carcinoma”, “lymph node positive”, “oligometastatic”, “multidisciplinary”, “atezolizumab”, “avelumab”, “nivolumab”, “pembrolizumab”, “erdafitinib”, “enfortumab vedotin”, and “sacituzumab govitecan”. English-language articles relevant to our pre-prepared questions were selected from the search results or identified from the reference lists of articles identified in the search, prioritizing the following article types: phase 2 and 3 clinical trials, guidelines, real-world studies, systematic reviews and meta-analyses, and narrative reviews (published within the previous 2 years). Relevant abstracts from the 2022 American Society of Clinical Oncology and European Society of Medical Oncology annual meetings were also included.

## Treatment Sequence

### Is There Any Evidence That a Multidisciplinary Approach Can Help the Management of Patients with Metastatic UC?

Treatment of UC that has spread beyond the regional lymph nodes mainly involves systemic therapy [4, 7, 15, 32, 33]. However, UC patients with distant metastases are a heterogeneous group that includes those with oligometastatic disease confined to a single organ or distant lymph nodes, who have a relatively favourable prognosis [13, 33, 34]. In a retrospective analysis of 128 patients with metastatic bladder cancer after total cystectomy, one-third of patients were identified as having oligometastatic disease, defined in this study as a solitary metastatic organ with up to three metastatic lesions (≤ 5-cm diameter) and no liver metastasis [33]. Optimal management of such patients is challenging because of the absence of clear treatment recommendations. Multidisciplinary team discussions are therefore important for selecting patients for metastasis-directed therapies to facilitate improved outcomes with systemic therapy in oligometastatic UC [13, 15, 32, 35–41]. Surgical metastasectomy is a rational approach for highly selected oligometastatic UC patients with adequate PS and metastases that can be completely resected [15, 32]. While retrospective evidence suggests that patients with a single, small (< 8 mm) lesion might gain the most benefit, those with more than one metastatic lesion, liver or bone metastases, primary UTUC or > 8-mm lesions are poor candidates for metastasectomy [32, 35]. When the suitability of 22 oligometastatic UC patients for metastasectomy was determined by a multidisciplinary team in a retrospective case series, and metastasectomy procedures were performed by experienced surgeons, with or without prior chemotherapy, encouraging survival outcomes (median OS 98.2 months) were reported [32].

Patients with oligometastatic UC may be inoperable because of a high risk of complications related to the site and dimensions of metastases [35, 41]. Metastasis-directed radiation therapy (MDRT) represents an alternative to metastasectomy for selected oligometastatic UC patients [37]. Evidence for MDRT is derived mainly from small retrospective studies, which involved mostly young bladder cancer patients with good PS and lymph node metastases after radical cystectomy. These studies have reported target tumour control rates of 57–100%, and median OS of 14.9–51.0 months [37]. There is a paucity of detail about the type of systemic therapy performed before, during or after MDRT, but preliminary evidence suggests that there may be synergistic and abscopal effects from combined ICI and MDRT [36, 37, 40, 42]. Emerging data suggest that MDRT may provide an opportunity to extend ICI therapy when there is progression at a limited number of metastatic sites [43].

#### Expert Opinion

Metastasectomy, MDRT, and systemic therapy should all be considered for UC patients with oligometastatic disease, with the choice and sequence of treatment guided by a multidisciplinary team of specialists, including urologists, radiation oncologists, oncologists, and pathologists. When assessing patient eligibility for metastasis-directed therapy, major considerations include patient PS and the number, size, and site(s) of metastases, as well as time from the initial diagnosis of primary UC.

### What Real-World Data Are Available on the Effectiveness and Tolerability of Maintenance Therapy with Avelumab?

On the basis of the results of the phase 3 JAVELIN Bladder 100 study [44••], avelumab is now standard of care for first-line maintenance treatment of advanced UC that has not progressed after 4–6 cycles of first-line platinum-based chemotherapy [7, 15, 45•]. In this study, 700 patients with unresectable locally advanced or metastatic UC and stable disease after chemotherapy with gemcitabine + cisplatin or carboplatin were randomised to avelumab + best supportive care (BSC) or BSC alone [44••]. After a median follow-up > 19 months, median OS was 21.4 months with avelumab + BSC versus 14.3 months with BSC alone (*p* < 0.001) [44••]. Exclusion of patients with progression during first-line chemotherapy may have contributed to the long OS with avelumab (the longest OS reported in a phase 3 trial in the first-line advanced UC setting) [44••, 45•]. Patients in the avelumab + BSC group also had longer PFS than patients receiving BSC alone (median PFS 3.7 vs 2.0 months). OS was significantly improved with avelumab, despite subsequent PD-1/PD-L1 inhibitor therapy in > 50% of patients with disease progression in the control arm, supporting a maintenance immunotherapy approach versus second-line treatment [44••]. Long-term follow-up (median ≥ 38 months) continued to show prolonged OS with avelumab + BSC versus BSC alone (median OS 23.8 vs 15.0 months, respectively) [46]. The OS benefits of avelumab maintenance therapy were seen irrespective of response (complete response, partial response, or stable disease) to first-line chemotherapy or the number of cycles of chemotherapy [47–49]. The avelumab safety profile was consistent with avelumab monotherapy in other cancer types [44••, 50]. Grade 3 immune-related adverse events (AEs) occurred in 7.0% of patients receiving avelumab, and 9.0% of these patients required high-dose corticosteroids [44••], reflecting some risk of serious immune-related AEs with avelumab maintenance therapy [51].

To date, real-world data on the tolerability and effectiveness of avelumab maintenance therapy in advanced UC patients are limited. However, outcomes after a median of 14.6 months’ follow-up in advanced UC patients eligible to receive first-line avelumab maintenance therapy as part of an Italian compassionate use programme involving 140 oncology centres were consistent with those of JAVELIN Bladder 100 [52]. Among 411 compassionate-use patients evaluable for OS and PFS, the 12-month OS rate was 69.2% (median OS not reached) and the 12-month PFS rate was 44.3% (median PFS 8.1 months). All-cause grade 3/4 AEs were reported in 7.1% of patients [52]. In addition, there is more real-world experience of avelumab treatment in other cancer types, providing some reassurance about its long-term safety profile [45•].

Future real-world studies in UC patients may provide insights into the utility of avelumab in patient populations under-represented in JAVELIN Bladder 100 (e.g. patients receiving platinum-containing regimens other than gemcitabine + cisplatin/carboplatin, and those with renal impairment or poor PS) [45•]. Long-term real-world studies may also help inform clinical judgement about the optimal duration of prior platinum-containing chemotherapy, the treatment-free interval between chemotherapy completion and avelumab initiation, and the duration of avelumab therapy [45•].

#### Expert Opinion

In addition to long-term clinical trial evidence from JAVELIN Bladder 100, preliminary real-world data provide support for avelumab as the standard of care first-line maintenance therapy for UC that has not progressed with platinum-containing chemotherapy, as confirmed by pre- and post-chemotherapy imaging. Based on this evidence, avelumab maintenance therapy should be started after 4–6 cycles of chemotherapy and a treatment-free interval of ≤ 10 weeks and thereafter administered until disease progression or unacceptable toxicity. Given that the benefits of avelumab appear to be independent of the number of chemotherapy cycles, the decision on how many cycles to administer should be based on the potential for benefit versus cumulative toxicity risk with chemotherapy. As there are no data indicating that six cycles provide greater survival benefits than four cycles, it is our opinion that four cycles should be adequate in most patients. Considering the low risk of cumulative toxicity with avelumab and chemotherapy, maintenance therapy should be started as soon as possible after the end of the last cycle and the assessment of tumour response to chemotherapy. There is currently no evidence available to support a fixed or maximum duration of avelumab therapy.

### What Are the Therapeutic Options and Outcomes in Patients Who Are Primarily Refractory to First-Line Treatments Using Platinum-Based Compounds?

Approximately 15–20% of patients with advanced UC progress on first-line platinum-based therapy and are therefore not eligible to receive avelumab first-line maintenance therapy [24, 44••, 53]. Second-line chemotherapies have demonstrated poor activity in these patients [7, 54].

Although there are no data specifically pertaining to primarily refractory patients, the PD-1 inhibitors pembrolizumab and nivolumab may be used as second-line therapy for patients who progress on platinum-based chemotherapy, based on evidence from phase 2 and 3 clinical studies in patients with progression after platinum-based chemotherapy (Table 1) [55, 56]. Currently, level 1 evidence exists only for pembrolizumab, which was associated with significantly prolonged OS versus docetaxel, paclitaxel, or vinflunine chemotherapy (median 10.4 vs 7.4 months; *p* = 0.002) in the phase 3 KEYNOTE-045 study [55]. Pembrolizumab is therefore the preferred standard of second-line therapy after platinum-based chemotherapy for advanced UC [7, 15]. Continued OS benefit and durable responses (> sixfold longer with pembrolizumab than with chemotherapy) were observed during 5 years of follow-up [57••]. Real-world study results were consistent with those of KEYNOTE-045, with a higher objective response rate (ORR) in the real-world setting (35.9% vs 21.1% in KEYNOTE-045) [55, 58].

**Table 1:** Summary of efficacy data from phase 2 and 3 clinical trials supporting the use of PD-1/PD-L1 inhibitors for the second-line treatment of platinum-refractory locally advanced or metastatic urothelial carcinoma [7, 15]

Agent	Trial	Phase	Comparator	N	Follow-up	OS	PFS	ORR	DOR
Pembrolizumab	KEYNOTE-045 [55, 57••, 115••]	3	Paclitaxel, docetaxel or vinflunine	542	Primary analysis				
					Median 14.1 months	Median 10.3 vs 7.4*	Median 2.1 vs 3.3 months	21.1% vs 11.4%**	Median NR vs 4.3 months
					Long-term follow-up				
					Median 27.7 months	Median 10.1 vs 7.3 months** 1-year 44.2% vs 29.8% 2-year 26.9% vs 14.3%	Median 2.1 vs 3.3 months 1-year 16.8% vs 6.2% 2-year 12.4% vs 3.0%	21.1% vs 11.0%	Median NR vs 4.4 months
					Median 62.9 months	Median 10.1 vs 7.2 months 3-year 20.7% vs 16.7% 4-year 11.0% vs 10.1%	4-year 9.5% vs 2.7%	21.9% vs 11.0%	Median 29.7 vs 4.4 months
Nivolumab	CheckMate 275 [56, 77]	2	Single-arm	270	Primary analysis				
					Minimum 9.0 (median 7.0) months	Median 8.9 months	Median 2.0 months	19.6%	Median NR
					Long-term follow-up				
					Minimum 33.7 months	Median 8.6 months 3-year 22.3%	Median 1.9 months	20.7%	Median 20.3 months

*p = 0.002

**p ≤ 0.001

*DOR*, duration of response; *NR*, not reached; *ORR*, objective response rate; *OS*, overall survival; *PD-1*, programmed cell-death protein-1; *PD-L1*, programmed cell-death ligand; *PFS*, progression-free survival

The phase 3 IMvigor211 study (*n* = 931) of the PD-L1 inhibitor atezolizumab versus docetaxel, paclitaxel or vinflunine chemotherapy in the platinum-treated advanced UC setting did not meet its primary endpoint of significantly improved OS for patients with high PD-L1 expression (median 11.1 vs 10.6 months; *p* = 0.41) [59]. Higher than expected OS with vinflunine may have contributed to this result. Although not formally tested for statistical significance, atezolizumab had a numerical OS advantage over chemotherapy in the overall population (median 8.6 vs 8.0 months; 12-month OS rates of 39.2% and 32.4%, respectively), and provided a prolonged duration of response versus chemotherapy (median 21.7 vs 7.4 months) [59]. Additionally, the SAUL study findings support the efficacy of atezolizumab in a large real-world population of patients with platinum-treated advanced UC [60•]. Although the totality of evidence from IMvigor211 and previous phase 2 data were promising, Roche Registration GmbH withdrew its application to the European Medicines Agency (EMA) for the use of atezolizumab as second-line treatment for metastatic UC after platinum chemotherapy in January 2021 for failure to meet their primary endpoint of OS [15]. Similarly, approval of durvalumab by the US Food and Drug Administration (FDA) has also been withdrawn based on negative results from the DANUBE trial [15, 59, 61].

Evidence from clinical trials in patients progressing after platinum-based chemotherapy and/or PD-1/PD-L1 inhibitors (Table 2) indicate that other potential options for platinum-refractory patients are enfortumab vedotin, sacituzumab govitecan, or erdafitinib (for patients with *FGFR2/3* alterations) [7, 15]. In the BLC2001 study with erdafitinib, the ORR was relatively high in patients previously treated with immunotherapy (59% vs 40% overall), potentially supporting the sequential use of PD-1/PD-L1 inhibitors before erdafitinib [62]. The phase 3 THOR study showed that erdafitinib significantly improved OS, PFS, and ORR versus investigator’s choice of chemotherapy in patients with FGFR alterations after prior treatment with PD-1/PD-L1 inhibitors, and that the toxicity of erdafitinib was consistent with its known safety profile [63]. In the global, open-label, phase 3 EV-01 study of patients with locally advanced or metastatic UC who had previously received platinum­-based chemotherapy and experienced disease progression during or after PD-1/PD-L1 inhibitor treatment, enfortumab vedotin significantly prolonged survival over standard chemotherapy [64•]. In the UNITE study, enfortumab vedotin had an ORR of 57% in patients with *FGFR3* alterations, and responses were observed in patients previously treated with erdafitinib, suggesting that these drugs can be used sequentially for patients with *FGFR3* alterations [65].

**Table 2:** Summary of efficacy data from phase 2 and 3 clinical trials of targeted therapies for previously treated and cisplatin-ineligible patients with locally advanced or metastatic urothelial carcinoma [7, 15]

Agent	Trial	Phase	Comparator	N	Treatment history	Follow-up	OS	PFS		ORR		DOR
Previously treated patients with locally advanced or metastatic urothelial carcinoma												
Enfortumab vedotin	EV-201 [116]	2	Single-arm	125	Platinum-containing chemotherapy and a PD-1/PD-L1 inhibitor	Median 10.2 months	Median 11.7 months	Median 5.8 months		44%		Median 7.6 months
	EV-301 [64•, 92]	3	Docetaxel, paclitaxel or vinflunine	608	Platinum-containing chemotherapy and a PD-1/PD-L1 inhibitor	Primary analysis						
						Median 11.1 months	Median 12.8 vs 8.9 months*	Median 5.5 vs 3.7 months*		40.6% vs 17.0%*		Median 7.3 vs 8.1 months
						Long-term follow-up						
						Median 23.75 months	Median 12.9 vs 8.9 months*	Median 5.6 vs 3.7 months*		NR		NR
Sacituzumab govitecan	TROPHY-U-01 [103]	2	Single-arm	113	Platinum-containing chemotherapy and a PD-1/PD-L1 inhibitor	Median 9.1 months	Median 10.9 months	Median 5.4 months		27%		Median 7.2 months
Erdafitinib	BLC2001 [67, 117]	2	Single-arm	99–101^a^	Chemotherapy (*n* = 88), immunotherapy (*n* = 22), no previous chemotherapy (*n* = 12)^b^ Number of treatments: 0 (*n* = 11), 1 (*n* = 45), ≥ 2 (*n* = 43)	Primary analysis						
						Median 11.1 months	Median 12.8 vs 8.9 months*	Median 5.5 vs 3.7 months*		40.6% vs 17.0%*		Median 7.3 vs 8.1 months
						Long-term follow-up						
						Median 24 months	Median 11.3 months 1-year 49% 2-year 31%	Median 5.5 months		40%		Median 6.0 months
Cisplatin-ineligible patients with locally advanced or metastatic urothelial carcinoma												
Atezolizumab	IMvigor210 [90]	2	Single-arm	119	Untreated	Median 17.2 months	Median 15.9 months	Median 2.7 months	23%		Median not reached	
Avelumab	ARIES [95]	2	Single-arm	71	Untreated	Median 10 months	Median 10.0 months 12-month 43%	Median 2.0 months	24%		NR	
Pembrolizumab	KEYNOTE-052 [89, 93]	2	Single-arm	370	Untreated	Primary analysis						
						Median 5 months	6-month 67%	Median 2 months 6-month 30%	24%		Median not reached	
						Long-term follow-up						
						Minimum 2 years	Median 11.3 months 12-month 46.9% 24-month 31.2%	Median 2.2 months 6-month 33.4% 12-month 22.0%	28.6%		Median 30.1 months	
Enfortumab vedotin	EV-201 [94]	2	Single-arm	89	Previous PD-1/PD-L1 inhibitors	Median 13.4 months	Median 14.7 months	Median 5.8 months 6-month 50% 12-month 33%	52%		Median 6 months	
Enfortumab vedotin + pembrolizumab	EV-103 [91•]	1b/2	Single-arm	45	Untreated	Median 20.0 months	Median 24.9 months	Median 12.3 months	73%		Median 25.6 months^c^	
	EV-103 Cohort K [92]	2	Enfortumab vedotin alone	149	Untreated	NS	NS	12-month 55.1% vs 35.8%	64.5% vs 45.2%		NR vs 13.2 months	

*p ≤ 0.001

^a^All patients had *FGFR2/3* alterations; after the clinical cut-off date for the primary analysis (*n* = 99), an additional 2 patients were enrolled, so *n* = 101 for the long-term follow-up

^b^Disease progression on or after ≥ 1 course of chemotherapy was required unless cisplatin-ineligible; immunotherapy was permitted

^c^Median follow-up 20.0 months

*DOR*, duration of response; *NR*, not reported; *NS,* not specified; *ORR*, objective response rate; *OS*, overall survival; *PD-1*, programmed cell-death protein-1; *PD-L1*, programmed cell-death ligand; *PFS*, progression-free survival

#### Expert Opinion

Despite the lack of evidence for this patient population, we agree that pembrolizumab is the most suitable second-line therapy. However, the decision to prescribe pembrolizumab should be considered carefully depending on the patient’s performance status and the possibility of rapid disease progression with platinum-based chemotherapy.

Based on evidence from phase 3 studies in patients treated with chemotherapy and immunotherapy, enfortumab vedotin is currently the preferred third-line therapy option, as well as erdafitinib in those harbouring *FGFR* alterations. Sacituzumab govitecan may also be considered as third-line treatment pending phase 3 trial results. Because no perspective data are available, when possible, we would encourage enrolling patients with progression as best response to platinum-based chemotherapy in clinical trials.

### What Is the Predictive and Prognostic Role of Tissue Markers in Epithelial Tumours?

*FGFR* alterations occur in up to 20% of patients with advanced UC [62, 66]. *FGFR3* alterations (mutations and fusions) are predictive markers for response to FGFR inhibitors (i.e. erdafitinib) [7, 62]. In the BLC2001 study, the ORR with erdafitinib was 49% in patients with *FGFR3* point mutations and 16% in those with *FGFR2/3* fusions, suggesting that patients with mutations may be more likely to respond to erdafitinib than those with fusions [67]. In clinical practice, the rate of *FGFR* testing is low (< 50%) in patients who have progressed after platinum-containing therapy [68]. Among patients tested in clinical practice, most (> 80%) undergo tissue-based FGFR testing; however, given tumour heterogeneity and evolutionary pressures during the course of treatment, blood-based testing may identify alterations not captured in archival primary tumour tissue [68–70].

High PD-L1 expression in urothelial tumour cells is associated with a poor UC prognosis [28, 71, 72]. The predictive value of PD-L1 expression for response to PD-1/PD-L1 inhibitors has been evaluated in many studies, although results were mixed and inconclusive [7, 73, 74•, 75]. Lack of standardisation for the detection of PD-L1 expression by immunohistochemistry, in which different antibody clones and cut-off values have been used in tumour cells and/or tumour-infiltrating cells, has hindered attempts to determine the role of PD-L1 as a predictive biomarker [74•, 75, 76]. A major limitation of PD-L1 staining relates to the significant proportion of PD-L1-negative patients who respond to immune checkpoint blockade [7]. The predictive value of PD-L1 was not confirmed in large phase 3 studies evaluating the integration of immunotherapy in the second-line setting for unresectable locally advanced or metastatic UC. The benefit of pembrolizumab appeared to be independent of PD-L1 expression on tumour cells and tumour-infiltrating immune cells [7, 55, 77]. Some evidence suggests that a composite biomarker approach (tumour mutational burden plus PD-L1) may help to identify patients who could benefit most from PD-1/PD-L1 therapy [77].

High microsatellite instability (MSI) is rare in UC, occurring in ~ 1% of UC tumours overall, but is considerably more common in Lynch syndrome-associated upper tract disease [4, 78]. Detection of high MSI could be useful to predict ICI response, and warrants consideration of early ICI therapy [79]. Although *NTRK* fusions occur very rarely in UC (0.1% of tumours) [78], their detection may prove to be similarly useful [80].

Nectin-4 expression has been shown to be consistently high in advanced UC tumours and represents an optimal target for an ADC [28, 29, 64•]. The association between the level of Nectin-4 expression and the response to enfortumab vedotin remains unclear, even if its evaluation is not required. A recent analysis has shown consistently high Trop-2 expression in UC tumour cells, including after prolonged exposure to enfortumab vedotin, suggesting feasibility of treatment with sacituzumab govitecan after enfortumab vedotin [28, 81].

HER2 has a relevant prognostic and predictive role in terms of response to targeted therapies in multiple types of cancer [82]. Recently, the role of HER2 has been investigated in UC, in which HER2 overexpression seems to have a prognostic role (i.e. correlated with muscle-invasive disease, recurrence, and shorter OS) [27, 82, 83]. Testing for HER2 expression in UC is not routine clinical practice, but literature to date suggests that up to approximately 40% of advanced UC patients are HER2 + (immunohistochemistry score of 2 + or 3 +) and may stand to benefit from HER2-targeted therapies [83]. HER-targeted ADCs (e.g. disitamab vedotin) are the most promising strategy in this field [84].

#### Expert Opinion

To help plan treatment, we recommend screening all patients with advanced UC for *FGFR3* mutations and *FGFR2/3* fusions. When available, blood-based liquid biopsy testing, using plasma-based next-generation sequencing, may be preferred to archival tumour biopsy tissue for molecular profiling. Although the predictive value of tumour or immune cell PD-L1 expression is unclear, it is known that second-line ICI therapy is an appropriate choice of treatment for patients with low PD-L1 expression because of the low efficacy of other treatment options and of tumour heterogeneity. However, platinum-based chemotherapy remains the best upfront option for patients with advanced UC, irrespective of PD-L1 expression. As such, we do not recommend testing for PD-L1 to determine eligibility for ICI therapy, unless it is being considered for patients unfit for platinum-based therapy in the first-line setting in Europe, as per the current indication for pembrolizumab [85]. In the USA, testing for PD-L1 is no longer required in the case of giving first-line pembrolizumab to patients unfit for platinum-based therapy [86]. Given the widespread expression of Nectin-4 and Trop-2 in UC, testing for expression of these biomarkers is not required to determine eligibility for treatment with enfortumab vedotin or sacituzumab govitecan.

### What Is the Optimal Treatment in Platinum-Unfit Patients (Both Cisplatin and Carboplatin)?

Data regarding the optimal treatment of advanced UC patients unfit for any platinum-based chemotherapy are limited, and these patients are often treated with BSC alone [7]. Alternative monochemotherapy regimens, including gemcitabine alone, may be an appropriate first-line treatment option for some patients who are not eligible for any platinum-containing chemotherapy [15, 87, 88].

Based on results of the single-arm phase 2 KEYNOTE-052, IMvigor210, and ARIES studies in cisplatin-ineligible patients (Table 2) [89, 90, 91•, 92–95], platinum-unfit patients can be considered for first-line immunotherapy (FDA approved irrespective of PD-L1 status, EMA approved only for PD-L1-positive patients) [7, 15]. However, these studies did not specify how many patients were unfit for any platinum-based therapy, and most patients did not respond to treatment, reflecting considerable unmet need in this group [7, 89, 90].

Studies of enfortumab vedotin have also been conducted in patients who were ineligible for cisplatin without specifying the proportion of patients unfit for platinum-based chemotherapy (Table 2). A clinically meaningful ORR and encouraging survival results were observed with enfortumab vedotin in the EV-201 study in patients who were ineligible for cisplatin and had received prior ICI therapy [94]. First-line combination therapy with enfortumab vedotin plus pembrolizumab has also shown promising antitumour activity in cisplatin-ineligible patients in the phase 1/2 EV-103 study, with a median duration of response (DOR) and OS of > 2 years [91•]. More recently, these data were corroborated by the phase 2 cEV-103 Cohort K study, which investigated enfortumab vedotin alone or in combination with pembrolizumab as first-line treatment in cisplatin-ineligible patients [96]. This combination is also being studied in the first-line setting versus platinum-based chemotherapy for platinum-eligible patients [91•].

#### Expert Opinion

Despite reliance on evidence in patients unfit for cisplatin-based chemotherapy, those who are unfit for any platinum-based chemotherapy (cisplatin or carboplatin), but still maintaining a sufficient performance status, should be offered first-line therapy with pembrolizumab (in Europe, limited to patients with PD-L1-positive status), while atezolizumab is no longer recommended for these patients. It is reasonable to consider monochemotherapy with gemcitabine or paclitaxel for selected platinum-unfit patients (especially in cases of PD-L1–negative tumours). It is also reasonable to offer enfortumab vedotin to platinum-unfit patients who do not respond to, or who progress on, immunotherapy. Enfortumab vedotin plus pembrolizumab is a promising first-line treatment that is currently under investigation compared with chemotherapy in the EV302 phase 3 study; because of its safety profile and metabolism, it may be considered a future option for cisplatin-ineligible patients.

## Management of Toxicity Linked to New Drugs

### When Should ADC or FGFR Inhibitor Treatment Be Suspended, and When Should It Be Re-started when Toxicity Is Resolved?

Although there are overlapping toxicities with new drugs for the treatment of UC, each agent has a unique profile with hallmark AEs [97, 98•].

Dose delays and reductions may be required for toxicities during enfortumab vedotin treatment, including hallmark AEs such as rash, peripheral neuropathy, hyperglycaemia, and pneumonitis. As Nectin-4 is expressed in epidermal keratinocytes, sweat glands, and hair follicles, dermatological events are common and anticipated treatment-related AEs with enfortumab vedotin [99]. The presentation of enfortumab vedotin–related dermatological AEs varies in distribution (localised or widespread), morphology, symptomatology, and severity, with onset generally occurring within the first treatment cycle. There is the potential for rare but severe and possibly fatal cutaneous adverse reactions, including Stevens-Johnson syndrome (SJS) and toxic epidermal necrosis (TEN). For grade 3 dermatological events, enfortumab vedotin should be withheld until improvement to grade ≤ 1 [100]. Even brief dose holds (e.g. 1 extra week “off” therapy at the end of a cycle) can be effective and lead to resolution of dermatological events [99]. Treatment can then be resumed at the same dose level, or with a dose reduction by one dose level at the discretion of the treating physician. Peripheral neuropathy, a known AE related to MMAE and other microtubule-disrupting agents, is a cumulative exposure event associated with enfortumab vedotin that needs to be managed carefully with treatment suspensions and/or dose reductions [51, 101]. Regarding enfortumab vedotin-induced hyperglycaemia (the aetiology of which is not fully understood) [97], treatment should be withheld in patients with blood glucose > 250 mg/dL [100]. Patients with non-specific pulmonary symptoms should be evaluated for pneumonitis, with enfortumab vedotin suspended or discontinued in cases of grade 2 or ≥ 3 pneumonitis, respectively [100, 102].

As well as being overexpressed in UC, low Trop-2 expression is present in normal cells, leading to a range of toxicities with sacituzumab govitecan [31]. Consistent with its irinotecan metabolite payload, the most common AEs with sacituzumab govitecan are nausea, diarrhoea, neutropenia, and fatigue [31, 97, 103]. These AEs are predictable and manageable, with the labelling information providing guidance on dose modifications and interruptions [104]. Neutropenia is the most likely cause of treatment delays and discontinuation during sacituzumab govitecan treatment [31, 103]. SN-38 is metabolized by uridine diphosphate-glucuronosyltransferase family 1 member A1 (UGT1A1)–mediated glucuronidation, and the risk of developing neutropenia is increased in patients homozygous for the UGT1A1*28 allele compared with heterozygous or wild-type patients [97, 103, 105, 106]. In all patients, grade 3–4 neutropenia should delay dosing until recovery to grade ≤ 1, but the dose should be reduced if recovery takes 2–3 weeks, and treatment discontinued if there is a delay of > 3 weeks for recovery. Grade 3–4 non-neutropenic haematological or non-haematological toxicity should also delay treatment until recovery to grade ≤ 1 and discontinued in the event of a 2- or 3-week recovery time.

Common FGFR inhibitor toxicities associated with erdafitinib include phosphate imbalances, diarrhoea, fatigue, and varied dermatological and ocular toxicities [67, 107]. The labelling information states that erdafitinib should be withheld when phosphorous levels are ≥ 7.0 mg/dL, and resumed after the level is reduced to < 5.5 mg/dL [108]. Although ocular AEs are common with erdafitinib, these events are mostly mild to moderate in severity and resolve with dose interruption and reduction [67]. For grade ≤ 3 ocular toxicities that resolve within 4 weeks, erdafitinib can be restarted at a lower dose [107, 108]. For grade 3 AEs other than hyperphosphataemia or central serous retinopathy/retinal pigment epithelial detachment, erdafitinib should be withheld until resolution to grade ≤ 1 [108].

#### Expert Opinion

It is vital that clinicians familiarise themselves with AEs that may occur with ADCs and targeted therapies. These AEs are often manageable with dose interruptions and/or reductions, including in patients receiving sacituzumab govitecan who are homozygous for UGT1A1*28.

### What Is the Utility of Corticosteroids in the Management of Toxicity of ADCs in UC?

Corticosteroids are used in the management of dermatological AEs associated with enfortumab vedotin [99, 109•]. Mild-to-moderate skin disorders can be treated with moderate potency topical corticosteroids [99]. Treatment is usually required for ≥ 4 weeks and may be utilised as-needed once the event has resolved or decreased by one grade of severity [99]. Oral corticosteroids (e.g. prednisone-equivalent 0.5 mg/kg/day for 14 days) are advisable for grade 3 events [99], although the use of systemic corticosteroids can cause enfortumab-associated hyperglycaemia [51].

Corticosteroids can be used prior to sacituzumab govitecan infusions in patients who have previously had infusion-related reactions [104]. Corticosteroids are also used to treat pneumonitis, which can occur during treatment with the ADC enfortumab vedotin [102, 110].

#### Expert Opinion

In conjunction with recommended dose interruptions and/or delays based on AE severity, topical and/or systemic corticosteroids can be used as part of supportive care to manage ADC toxicities, such as dermatological AEs and pneumonitis. Prompt recognition and management of such events with corticosteroids may allow for continued ADC therapy.

### What Are the Methods to Prevent ADC Toxicity?

Patients receiving enfortumab vedotin must be advised of the potential for serious skin reactions, common symptoms, and the importance of early reporting [111]. Patients with a history of dermatological conditions (i.e. psoriasis), rash/pruritus, allergies, dry skin, immunosuppression, and/or high sun exposure may be predisposed to skin reactions [111]. Prophylactic strategies include the use of barrier-protecting agents (e.g. zinc-containing moisturizers), especially in intertriginous areas like the axillae and groin; fragrance-free, gentle emollients (e.g. white petrolatum) at least twice daily; and protection from ultraviolet radiation with sunscreen on exposed skin [99, 111]. For patients with a skin reaction, full-body examination helps ensure accurate estimation of the affected body surface area [111]. Treating mild skin reactions allows for more rapid and effective management, potentially preventing development of severe skin reactions that could lead to treatment delays [111]. Early referral to a dermatologist for lower-grade skin reactions is also a reasonable approach for proactive evaluation and management [111].

Early detection and management of enfortumab vedotin-associated peripheral neuropathy may allow for some recovery from symptoms and prevent symptoms worsening [111]. However, in our experience, patients can downplay the extent of peripheral neuropathy symptoms to avoid treatment delay or discontinuation. Clinicians must ensure that patients are aware of the signs and symptoms of peripheral neuropathy, and the importance of prompt reporting and management to reduce risk of severe and potentially irreversible symptoms [111].

Educating patients about the potential for hyperglycaemia with enfortumab vedotin, the importance of recognising and reporting symptoms, and the potential for serious complications is also essential. Counselling on lifestyle modifications, such as a healthy diet that is low in simple carbohydrates, regular exercise, and weight loss (if indicated), is advisable [111]. Routine monitoring of non-fasting blood glucose is recommended prior to each enfortumab vedotin dose [111].

It is important to carefully monitor patients receiving sacituzumab govitecan who are known to be homozygous for UGT1A1*28 [97]. Prophylactic dose modifications and granulocyte colony-stimulating factor (G-CSF) support should be considered in patients known to be homozygous for UGT1A1*28. However, as a substantial proportion of UGT1A1*28 homozygous patients do not experience grade ≥ 3 neutropenia, the management of neutropenia is considered more appropriate than prophylactic screening for patients with the *UGT1A1* genotype [103, 105]. G-CSF support, although not routinely recommended as primary prophylaxis of neutropenia, may be considered on a case-by-case basis (i.e. for frail patients). G-CSF can be considered as secondary prophylaxis [104].

For prevention of sacituzumab govitecan–induced nausea and vomiting, premedication (e.g. dexamethasone with a serotonin 5-HT3 receptor antagonist) is recommended before each sacituzumab govitecan dose [104]. Premedication with antipyretics and histamine (H) 1/H2 blockers is also recommended for prevention of infusion-related reactions [104].

#### Expert Opinion

ADC therapy can be optimised through patient and caregiver education, proactive patient monitoring, early identification of AEs, and timely intervention to alleviate symptoms, thereby reducing the need to suspend or discontinue treatment.

### Can an Involvement of Several Specialists (Oncologists Plus Other Specialists) Be Hypothesised in the Prevention and Management of AEs?

Patients receiving ADCs or erdafitinib may benefit from a multidisciplinary approach similar to that proposed for the optimal management of other cancer therapy-induced AEs [112]. Specialists that may help in the prevention and management of AEs regularly encountered with ADCs and targeted therapy include dermatologists, endocrinologists, ophthalmologists, and pulmonologists.

For skin toxicities (i.e. with erdafitinib or enfortumab vedotin), dermatology referral is recommended for patients with grade 3 or intolerable grade 2 skin toxicities that have not responded to ≥ 4 weeks of therapy [99, 107]. Pre-emptive referral to a dermatologist is also advisable in patients scheduled to receive erdafitinib or enfortumab vedotin who have a history of skin conditions, particularly autoimmune skin diseases.

For patients receiving erdafitinib or enfortumab vedotin, an ophthalmology consultation is advised in the event of vision changes, such as blurry vision or floaters [107, 111]. Erdafitinib recipients with blurry vision would benefit from monthly ophthalmology evaluations for early diagnosis and management of serous retinopathy [107].

Patients with pre-existing diabetes or hyperglycaemia should be monitored closely by an endocrinologist during enfortumab vedotin therapy [111]. Endocrinologist referral should also be considered for patients at risk for diabetes.

Interstitial pneumonitis, which can lead to respiratory failure, often presents insidiously with dyspnoea, dry cough, mild fever, and fatigue among the first clinical symptoms [110]. Differentiating enfortumab vedotin-induced pneumonitis from other causes of pneumonia, followed by an appropriate course of treatment, is therefore essential [102, 110], and best performed by a pulmonologist.

A multidisciplinary approach to geriatric assessment can also help to determine whether frail, elderly patients are fit enough to tolerate treatment, and to minimise AEs [19, 113]. Comprehensive geriatric assessment should be conducted to identify age-specific risk factors and determine patient frailty [19].

#### Expert Opinion

The involvement of different medical specialties with in-depth knowledge of the specific organs or physiological systems affected by an AE is important to help patients with limited treatment options to comply with treatment and derive maximum benefit from treatment with ADCs or targeted drugs.

## Algorithm and Clinical Response

### When Should a Patient Be Re-staged After First-Line Platinum-Based Treatment?

Platinum-based chemotherapy can be continued for a maximum of six cycles, depending on response and toxicity [15]. JAVELIN Bladder 100 data support the administration of avelumab in patients without disease progression after 4–6 chemotherapy cycles [45•].

#### Expert Opinion

Given that eligibility for avelumab should be assessed after 4–6 chemotherapy cycles, computed tomography scanning should be performed after four cycles of platinum-based chemotherapy, and, if chemotherapy is not stopped at four cycles, again after six cycles. Re-evaluation is advised after 2–3 cycles in patients with suspected early disease progression.

### What Therapeutic Algorithm Should Be Followed in Locally Advanced or Metastatic UC and How Should It Evolve in the Light of Novel Treatment Options?

**Fig. 1:**
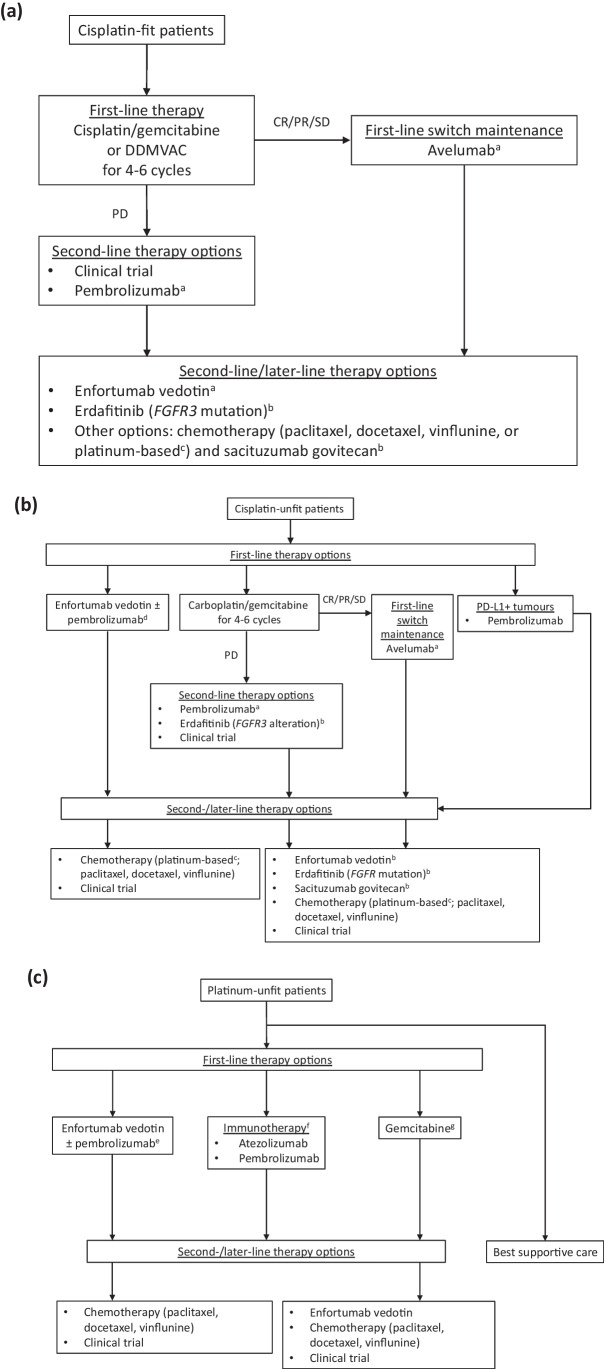
Treatment algorithm for **a** cisplatin-fit patients, **b** cisplatin-unfit patients, and **c** platinum-unfit patients with advanced urothelial carcinoma. *CR*, complete response; *DDMVAC*, dose-dense methotrexate, vinblastine, doxorubicin, and cisplatin; *PD*, progressive disease; *PR*, partial response; *SD*, stable disease. ^a^Level 1 evidence after chemotherapy + immunotherapy; ^b^FDA-approved based on phase 2 clinical trial data in patients pre-treated with platinum-based therapy + immunotherapy; ^c^if > 1 year after first-line cisplatin-based chemotherapy; ^d^likely future treatment option based on recent phase 2 clinical trial data (currently off-label); ^e^potential off-label pathway for some patients based on recent phase 2 clinical trial data in cisplatin-unfit patients; ^f^approval (FDA, regardless of PD-L1 status: EMA, PD-L1 + only) based on phase 2 clinical trial evidence in cisplatin-unfit patients; ^g^appropriate option for PD-L1-negative patients

Figure 1 summarises the treatment algorithms for unresectable locally advanced or metastatic UC based on the evidence already discussed in this article.

Now that avelumab maintenance therapy is approved specifically for patients not progressing on first-line platinum-based chemotherapy, it is especially important to minimise the risk of progression during first-line platinum-based chemotherapy by striving to offer as many patients as possible the opportunity to receive first-line cisplatin, given its superiority to carboplatin-based treatment [7, 13]. Nephrology consultations and various nephrotoxicity mitigation strategies may be used to facilitate use of cisplatin in patients with glomerular filtration 40–60 mL/min, who would be ineligible for cisplatin according to Galsky criteria alone [13].

In addition to being used as first-line treatment, platinum-based chemotherapy is used as neoadjuvant and adjuvant therapy, and many ongoing studies are investigating immunotherapy in this setting [7]. It is therefore expected that an increasing number of patients with metastatic UC will have already received platinum and/or immunotherapy agents as neoadjuvant or adjuvant treatment [7]. If ≥ 12 months has elapsed since the end of neoadjuvant or adjuvant treatment, the same first-line treatment as treatment-naïve patients can be used [7]. Otherwise, later-line treatment options would be recommended.

Based on evidence from clinical studies in patients previously treated with platinum-based chemotherapy and PD-1/PD-L1 inhibitors (Table 1) [64•, 67, 103], which included patients treated with adjuvant or neoadjuvant platinum chemotherapy with disease recurrence within 12 months of treatment completion and subsequent progression on immunotherapy, the third-line treatment options are enfortumab vedotin, sacituzumab govitecan, and erdafitinib (Fig. 1). In addition, these drugs are second-line therapy options after progression on first-line avelumab maintenance therapy (Fig. 1) [7, 15]. Enfortumab vedotin clinical study data were replicated in the real-world UNITE study [65]. Real-world erdafitinib OS is also in line with clinical study results [68]. Sacituzumab govitecan research is not as far advanced as the other two agents, with no available real-world data thus far.

Regarding chemotherapy re-challenge after prior platinum-based therapy, disease control appears most likely when patients have achieved disease control with prior platinum therapy, and a longer time has elapsed since their prior platinum treatment, as illustrated in the Retrospective International Study of Cancers of the Urothelium (RISC) study [114]. Re-challenge with platinum-based chemotherapy resulted in better OS and disease control than non-platinum-based chemotherapy [114].

#### Expert Opinion

After progression on first-line avelumab maintenance therapy, patients should be offered an alternative to immunotherapy. We recommend enfortumab vedotin (or erdafitinib for patients with *FGFR2/3* alterations), which is currently the only agent with level 1 evidence in patients previously treated with chemotherapy and PD-1/PD-L1 inhibitors, albeit in the third-line setting. Accordingly, enfortumab vedotin is recommended as later-line therapy in patients who have received first-line platinum and second-line immunotherapy, including patients recently treated with platinum-based chemotherapy in the neoadjuvant or adjuvant setting. Additionally, enfortumab vedotin is a first- or second-line treatment option in platinum-unfit patients, although a larger body of evidence for first-line immunotherapy in cisplatin-ineligible patients means that this is the preferred first-line option for platinum-unfit patients. Platinum-fit patients who progress after receiving maintenance avelumab or later-line therapy may be considered for platinum re-challenge. When possible, enrolling patients in clinical studies is encouraged.

## Conclusion

The therapeutic landscape for advanced UC has become considerably more diversified in recent years. First-line avelumab maintenance therapy is a new standard of care for patients who do not progress on platinum-based chemotherapy, and ADC and FGFR-targeted therapy provide welcome new treatment options in the post-platinum/ICI setting. However, proven therapeutic options for patients unfit for platinum-based chemotherapy remain an unmet need. The optimal sequence of novel agents also remains to be determined and the search continues for predictive biomarkers that may aid in treatment selection and sequencing. The optimal sequence and potential combinations of novel agents are the subject of on-going studies that will hopefully answer these outstanding questions. In the meantime, multidisciplinary collaboration and expert opinion can help to optimise the use of novel agents within existing treatment algorithms.

## Data Availability

Data sharing is not applicable to this article as no new data were created or analysed.
